# Analysis of Nirmatrelvir Entry into Pulmonary Lining Fluid in Patients with COVID‐19: A Unique Perspective to Explore and Understand the Target Plasma Concentration of 292 ng/mL in Antiviral Activity

**DOI:** 10.1002/iid3.70075

**Published:** 2024-11-15

**Authors:** Wenjing Zhang, Lin Xia, Zhilong Yuan, Yang Jiao, Zhuo Wang

**Affiliations:** ^1^ Department of Pharmacy, Shanghai Changhai Hospital The First Affiliated Hospital of Naval Medical University Shanghai China; ^2^ School of Pharmacy Bengbu Medical University Bengbu China; ^3^ Department of Respiratory and Critical Care Medicine Shanghai Changhai Hospital, The First Affiliated Hospital of Naval Medical University Shanghai China

**Keywords:** concentration, COVID‐19, human epithelial lining fluid, nirmatrelvir

## Abstract

**Background:**

Currently, the applicant has chosen a target plasma trough concentration for nirmatrelvir, which is adjusted to 292 ng/mL based on the drug's molecular weight (499.54 Daltons), its binding to human plasma proteins (69%), and the in vitro antiviral EC_90_ value (181 nM). However, the current exposure‐effect relationships (ER) analysis of viral load in patients enrolled in the EPIC‐HR study has not revealed clinically significant trends in the ER. Given that the lungs are the primary site of COVID‐19 infection, we aim to further understand this exposure relationship by exploring and analyzing the penetration characteristics of nirmatrelvir in the lungs.

**Objectives:**

To explore and understand the target plasma concentration of 292 ng/mL in antiviral activity.

**Methods:**

We identified all critically ill patients with coronavirus disease 2019 who received nirmatrelvir/ritonavir treatment in the respiratory intensive care unit of Changhai hospital between January 2023 and October 2023. Samples of plasma and bronchoalveolar lavage fluid were obtained at pre‐dose trough concentrations after administration of nirmatrelvir (NMV). The relationship between NMV levels in plasma and the epithelial lining fluid (ELF) was assessed by determining concentrations of NMV.

**Results:**

There was a significant relationship between NMV levels in plasma and ELF (ELF = 0.4976*PLASMA‐ 204; *R* = 0.96), with a correlation whose slope (0.4976) suggested that the blood‐to‐ELF ratio of drug penetration was 2:1. A negative value from the equation indicates that NMV requires to reach baseline concentration to penetrate the ELF.

**Conclusions:**

The relationship between NMV levels in plasma and ELF with low permeability and a negative baseline value suggests that the target plasma concentration of 292 ng/mL is insufficient for antiviral activity. This study provides a unique perspective to explore and understand no clinically meaningful trend of exposure‐response relationships in patients enrolled in EPIC‐HR.

## Graphical abstract

This study is the first to explore nirmatrelvir penetrating human epithelial lining fluid from the blood. We assessed the relationship between nirmatrelvir levels in plasma and those in human epithelial lining fluid to elucidate that the currently applied nirmatrelvir target plasma exposure of EC90 value (292 ng/mL) is not adequate for patients.

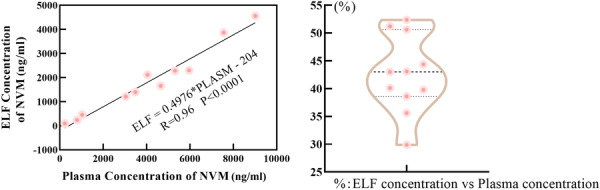

## 1 Introduction

Severe acute respiratory syndrome Coronavirus 2 (SARS‐CoV‐2), the causative agent of the coronavirus disease 2019 (COVID‐19), is an important member of the coronavirus family [1, 2]. Oral nirmatrelvir (NMV) tablets co‐packaged with ritonavir tablets are an important therapeutic option for patients, exhibiting a broad‐spectrum activity across the Coronaviridae family. The SARS‐CoV‐2 infects cells through the angiotensin‐converting enzyme 2 receptors, with the lung and bronchial epithelial cells being the primary sites of infection [3]. However, the information regarding the disposition of NMV in the epithelial lining fluid (ELF) is limited or even lacking [4].

In cell cultures, nirmatrelvir exhibited antiviral activity against SARS‐CoV‐2 infection in differentiated normal human bronchial epithelial cells with 90% effective concentration (EC_90_) values of 181 nM, as measured by viral replication after 3 days of nirmatrelvir exposure. Therefore, the applicant selected a nirmatrelvir target plasma trough concentration of NMV above the in vitro antiviral EC_90_ [292 ng/mL; adjusted by nirmatrelvir molecular weight (499.54 Daltons) and human plasma protein binding (69%)] [4], which is believed to be essential for a pharmacodynamic response [4, 5]. Although these studies in vitro models and clinical trials have dramatically expanded our knowledge of exposure‐effect relationships (ER) with NMV, no clinically meaningful trend in exposure‐response relationship was evident based on the current ER analysis of viral load in patients enrolled in EPIC‐HR. Here, we detect and analyze the relationship between bronchoalveolar lavage fluids and plasma, which provides a unique perspective to explore and understand the exposure relationship of NMV between plasma and ELF concentrations. This study may not only partially explain current ER analysis from the primary sites of infection, but also speculate that the free NMV concentration in the ELF with EC_90_ value needed for the corresponding plasma concentration was > 2 × EC_90_ of plasma and ＜ 3.4 × EC_90_ of plasma, which will have the potential to improve the clinical use and therapeutic outcomes of oral nirmatrelvir tablets.

## 2 Methods

### 2.1 Study Design and Setting

The study had a noninterference policy, and any treatment decisions about patients in the study were at the discretion of the treating physicians, which was approved by the Shanghai Changhai Hospital Ethics Committee (CHEC 2023‐100). In addition, patients needed to meet several criteria for the following inclusion: patients with the coronavirus disease 2019 (COVID‐19) [6] received nirmatrelvir/ritonavir treatment; blood and bronchial lavage fluid (BALF) samples were obtained from clinicians in the course of routine clinical care, which were collected at pre‐dose trough concentrations after administration of NMV; bronchoalveolar lavage was collected within 1 h after blood samples; All patients were required to provide oral informed consent and be at least 18 years old. A total of 7 patients were eligible during a ten‐month study period between January 2023 and October 2023. Detailed data on treatment history, treatment patterns, and outcomes were collected. (See Table S1 and Figure S1).

### 2.2 Determination of Nirmatrelvir in Human Plasma and Bronchoalveolar Lavage Fluid by Liquid Chromatography Coupled to Tandem Mass Spectrometry

Procedures for determining NMV in biological samples have been previously published [7, 8]. The system consisted of high‐performance liquid chromatography (model 20AD; Shimadzu, Kyoto, Japan) with a QTrap hybrid quadrupole linear ion trap mass (model AB Sciex3200; Foster City, CA, USA). The NMV was detected in the positive ion mode using multiple reaction monitoring of the transitions at m/z 500.2 → 319.3. A gradient mobile phase consisting of Mobile phase‐A: 0.1% formic acid solution and Mobile phase‐B: acetonitrile was employed. Isocratic elution was performed with 20% mobile phase A (0.1% formic acid in water) for a total run time of 8 min. The prepared sample was pumped through a Zorbax SB‐Aq C_18_ column (250 × 4.6 mm I.D., 5 μm particle size) at a flow rate of 1.0 mL·min^−1^ at 30℃ of column temperature.

### 2.3 Calculated NMV in the ELF by Urea as an Endogenous Marker

Since urea diffuses readily throughout the body, the urea concentrations in ELF and plasma are the same. We used urea as an endogenous marker of ELF dilution to quantify the apparent epithelial lining fluid concentration (C_ELF_), as described previously [9]: Concentration in ELF = Concentration in BALF × Urea in serum/Urea in BALF. The urea content of BALF samples was determined by using routine biochemical tests.

### 2.4 Samples Processing

Blood samples: The blood samples were collected for routine laboratory tests, which were separated into two tubes and then centrifuged for plasma and serum. The plasma was collected for our testing (determination of nirmatrelvir concentration). The urea in serum was performed in the routine laboratory.

Bronchoalveolar lavage fluid: 10 mL aliquots of 0.9% normal saline were instilled into the area of pneumonia, with the fluid being immediately aspirated and placed on ice after each aliquot. The sample was sent to the laboratory and immediately centrifuged at 10,000 g for 5 min. The supernatant was separated into two independent samples for determining urea and nirmatrelvir concentrations, which were collected and frozen until the assays could be performed.

### 2.5 Statistical Analysis

All statistical analyses and graphing were performed using GraphPad Prism 8. Correlational analyses were performed using Pearson's correlation analysis to further examine the relationship between the plasma concentration of nirmatrelvir and its concentration in the epithelial lining fluid.

## 3 Results

### 3.1 Baseline Characteristics of Study Subjects

Seven patients (male) with severe acute respiratory disease coronavirus 2 were enrolled in this study (22 samples in total, including 11 blood samples and 11 BALF samples). Six patients were classified as critically ill with being not administered orally and given drugs through a nasal cannula. The glomerular filtration rate was calculated: 2/7 （28.5%）of the patients had kidney damage with increased GFR (eGFR> 120 mL/min/1.73 m^2^), one patient (14.3% of all patients) had a normal function (eGFR ≥ 90 and < 120 mL/min/1.73 m^2^), and four patients (57.1% of all patients) had a moderate impairment (estimated glomerular filtration rate [eGFR] ≥ 30 and < 60 mL/min/1.73 m^2^). The mean ± SD age and weight of the subjects were 83.29 ± 10.72 years and 62.71 ± 12.71 kg, respectively. Further details can be found in Table S1, and the treatment course and therapeutic drug monitoring results of nirmatrelvir/ritonavir in plasma and BALF are shown in Figure S1.

### 3.2 Precision and Accuracy of the Analytic Method

The assays were linear (*R* = 0.96) over the 0.05–10 mg/mL range for plasma and 0.02–4 mg/mL for the BALF. The intra‐day and inter‐day quality control samples had a coefficient of variation of 8.1% for all matrixes.

### 3.3 The ELF and Plasma Concentration of Nirmatrelvir and Correlation Analysis

**Figure 1:**
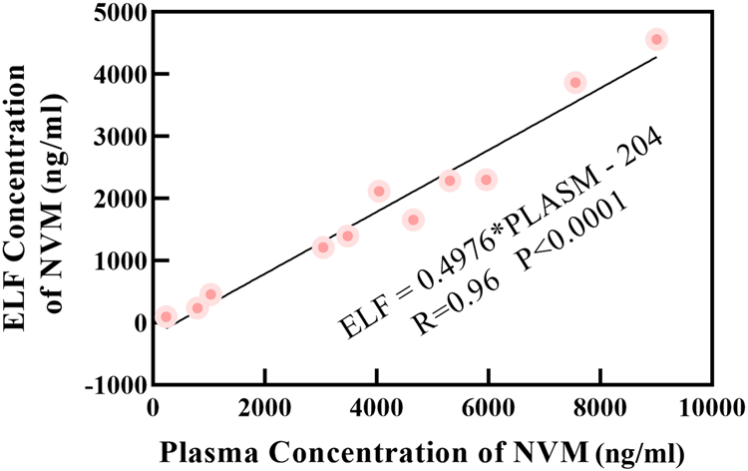
The relationship between nirmatrelvir concentrations in plasma and ELF.

Detailed information data on urea, albumin, and nirmatrelvir levels in plasma, BALF, and ELF of the seven patients were presented in Table 1. A scatterplot with the associated linear regression line of the ELF versus plasma nirmatrelvir concentrations is shown in Figure 1 and observed good linear relationships (ELF = 0.4976*PLASMA‐204; *R* = 0.96, *p* < 0.0001).

**Table 1:** Levels of urea, albumin, and Nirmatrelvir in plasma, bronchoalveolar lavage fluid (BALF), and epithelium lining fluid (ELF) of the seven patients.

Patient No.	Urea _serum_	Urea _BALF_	C_BALF_ (ng/mL)	C_ELF_ (ng/mL)	C plasma (ng/mL)	C_ELF_/C plasma	Albumin in plasma (g/L)
1 (round 1)	19	2.7	65	457	1030	44.37 (%)	40
1 (round 2)	13.7	2.1	334	2288	5310	43.09 (%)	38
2	32.5	4.6	326	2302	5960	38.62 (%)	29
3	20.7	3.4	750	4560	9010	50.6 (%)	25
4	13.2	1.7	156	1211	3040	39.8 (%)	32
5	19.1	3.0	608	3867	7550	51.2 (%)	30
	29.5	4.0	236	1658	4650	35.6 (%)	32
	26.6	3.6	32	236	790	29.9 (%)	31
6	11.7	1.7	308	2119	4040	52.4 (%)	33
7	8.3	1.2	202	1397	3480	40.1	39
	8.8	1.4	16.5	99	230	43.0	39

**Figure 2:**
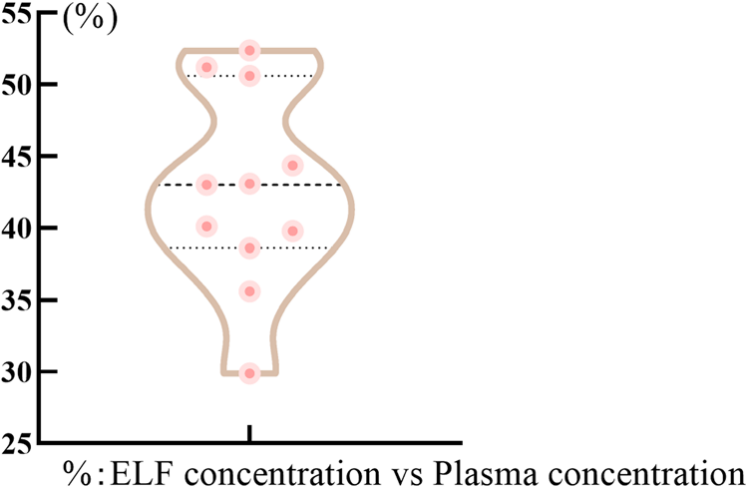
The penetration of nirmatrelvir in pulmonary epithelial intimal fluid.

The ELF: plasma ratios (RELF:P) of nirmatrelvir were calculated using the plasma as reference (C_ELF_/C_plasma_) for each sample. As shown in Figure 2, the ELF: plasma concentration RELF: P of nirmatrelvir was in the median range of 0.426 ([0.299–0.524], *n* = 11).

## 4 Discussion

Some studies have found that a single ELF or plasma concentration point can be substituted for the time curve to calculate permeability [10, 11]. In addition, the fluctuation of drugs within 1 h in trough concentrations is small‐scale. For the reasons mentioned above, and based on our study's noninterference policy, we compared concentrations (e.g., ELF and plasma concentrations) obtained under the same administration within 1 h to estimate penetration ratios.

### 4.1 The Concentrations of NMV in Pulmonary Epithelial Lining Fluid and Plasma

From Figures 1 and 2, the permeability of lung tissue was low (RELF:P ranged from 0.299 to 0.524), but increasing NMV concentrations in the blood will lead to an increase in pulmonary ELF levels. There was a significant relationship (Range: 230–9010 ng/mL) between NMV levels in plasma and those in ELF (ELF = 0.4976*PLASMA‐204;R = 0.96, *p* < 0.0001), with a correlation whose slope (0.4976) suggested that the blood‐to‐ELF ratio of drug penetration was 2:1.

### 4.2 The Analysis of ELF Concentrations and Plasma Exposure

According to our linear equations, a negative value indicates that penetration of NMV through blood vessel walls into ELF is required to achieve a baseline concentration. Hence, we can predict that a plasma target concentration of 292 ng/mL corresponds to an ELF concentration that can not achieve the EC_90_ value. COVID‐19 releases inflammatory mediators such as IL‐1β, IL‐6 and leukotrienes [12], further amplifying inflammation and disrupting the air‐blood barrier [13], which can contribute to increased drug permeability. The degree of permeability is correlated with the severity of lung damage. Indeed, some studies have employed the measurement of albumin concentrations in bronchoalveolar lavage fluid as a means to assess the magnitude of alveolar injury [14]. Notably, NMV with low solubility (0.90–1.21 mg/mL throughout the physiological pH range) and with low permeability of NMV in vitro [15], make it challenging for them to cross the cell membrane. This implies that patients with mild‐to‐moderate have a lower NMV penetration compared with severe patients.

Pharmacodynamics for NMV targeting respiratory pathogens (COVID‐19) should be based on free rather than total drug concentrations in ELF. According to the linear equations (ELF = 0.4976*PLASMA‐204; *R* = 0.96, *p* < 0.0001), ELF values of 181 nM (90 ng/mL) need plasma concentration values of 591 ng/mL (approximately 2 × EC_90_), and ELF values of 292 ng/mL need plasma concentration values of 996 ng/mL (approximately 3.4 × EC_90_). In previous studies [9, 16], small amounts of plasma proteins normally reach the alveolar epithelial surface. A value very similar to the average albumin concentration in the ELF was 12% of its level in plasma [9, 16]. Due to the inability to obtain the protein binding rate of NMV in ELF, we can only speculate that the free NMV concentration in the ELF with the EC_90_ value needed for the corresponding plasma concentration was > 2 × EC_90_ and ＜ 3.4 × EC_90_. Interestingly, our hypothesis could explain why the viral clearance rates were higher on the Day 5 nirmatrelvir minimum plasma concentration (> 3×) EC_90_ plasma concentration in patients enrolled in EPIC‐HR [4, 5]—this concentration ensures that the ELF concentration of nirmatrelvir also reaches or above the EC_90_, and was similar on the Day 5 nirmatrelvir minimum plasma concentration (3–5×) EC_90_ plasma concentration and (> 5x) EC_90_ enrolled in EPIC‐HR. The hypothesis can also explain why the viral clearance rates were similar on the Day 5 nirmatrelvir minimum plasma concentration (1–3×) EC_90_ plasma concentration and placebo group enrolled in EPIC‐HR. This was because a plasma concentration in this range may not achieve the EC_90_ in the epithelial lining fluid, leading to a suboptimal antiviral effect and comparable viral clearance rates to the placebo group. Noted, there is no contradiction between our hypothesis and the nirmatrelvir treatment shows significant clinical efficacy. The data from EPIC‐HR demonstrated that a significant portion (93.8%) of available C_trough_ values were > 3 × EC_90_.

The main limitation of this study was the small sample size, and the inclusion of participants from only a single center, which might restricted outcomes. In the future, increasing the number of data to verify the feasibility and relationship between NMV levels in plasma and those in ELF, may improve clinical outcomes.

## 5 Conclusion

Overall, the relationship between NMV levels in plasma and human epithelial lining fluid, with low permeability and a negative baseline value, suggests that the target plasma concentration of 292 ng/mL is insufficient for antiviral activity. Consequently, the currently applied NMV target plasma exposure of 292 ng/mL needs further studies to validate the clinical response. This study provides a unique perspective to explore and understand no clinically meaningful trend of exposure‐response relationships in patients enrolled in EPIC‐HR.

## Author Contributions

**Wenjing Zhang:** data curation, formal analysis, funding acquisition, investigation, validation, writing–original draft, writing–review and editing. **Lin Xia:** formal analysis, investigation, project administration, supervision, validation, visualization, writing–review and editing. **Zhilong Yuan:** software, supervision, visualization. **Yang Jiao:** conceptualization, methodology, project administration, supervision. **Zhuo Wang:** conceptualization, funding acquisition, methodology, project administration.

## Conflicts of Interest

The authors declare no conflicts of interest.

## Supporting information

Supporting information.

Supporting information.

## Data Availability

The authors have nothing to report.
