# Supplementary material for: Analysis of Nirmatrelvir Entry into Pulmonary Lining Fluid in Patients with COVID‐19: A Unique Perspective to Explore and Understand the Target Plasma Concentration of 292 ng/mL in Antiviral Activity
Source: Immun Inflamm Dis. 2024 Nov 15;12(11):e70075. doi: 10.1002/iid3.70075 (PMC11565441; doi:10.1002/iid3.70075)
Supplement: Supplementary file 1 — Supporting information. [file IID3-12-e70075-s001.docx]

**
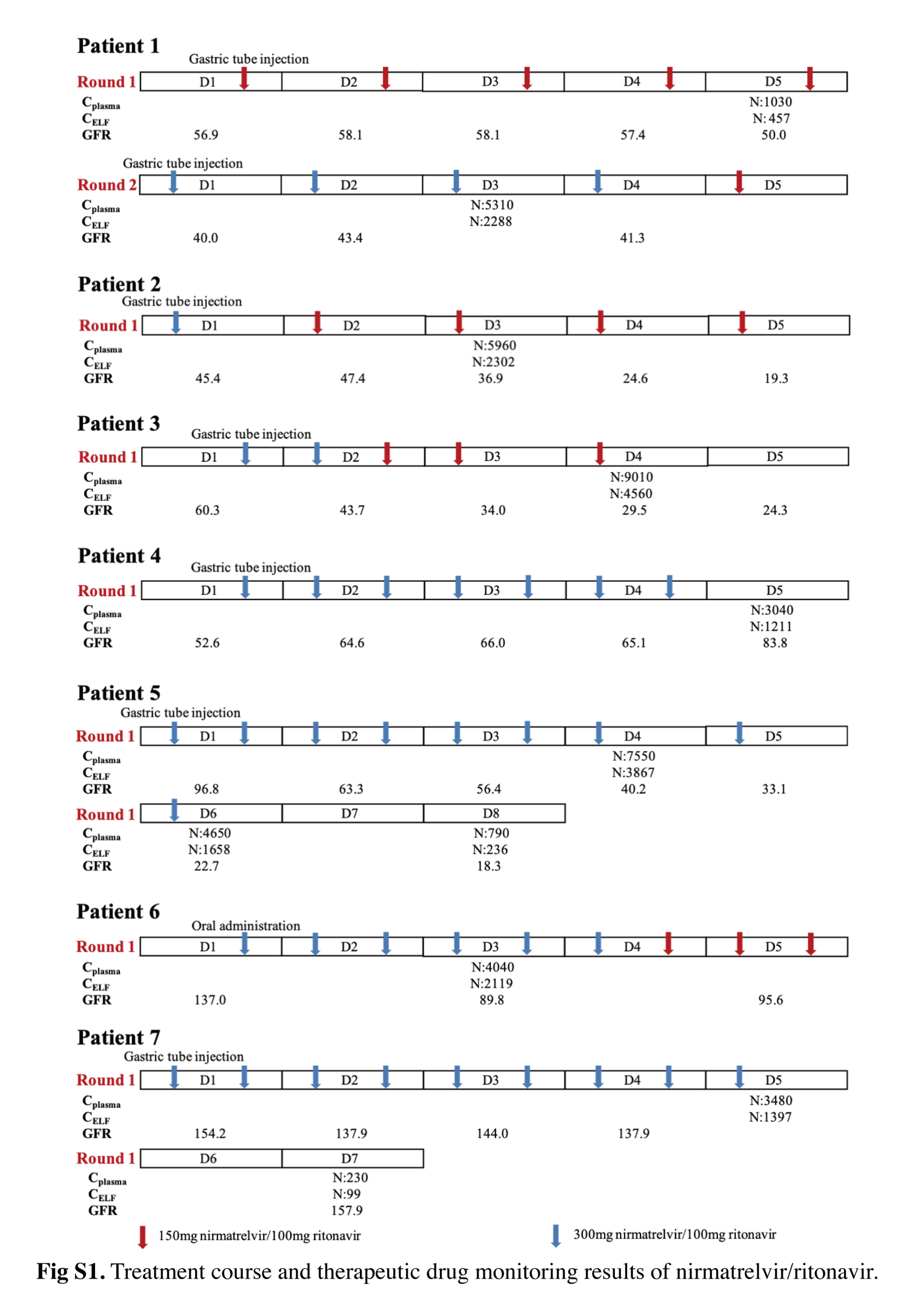
Supplementary Fig 1. Treatment course and therapeutic drug monitoring results of nirmatrelvir/ritonavir**

Note: C_plasma_: Plasma Concentration (ng/mL) C_ELF_: Epithelial Lining Fluid Concentration (ng/mL)

GFR: Glomerular Filtration Rate (mL/min/1.73m^2^)
