# Supplementary material for: Analysis of Nirmatrelvir Entry into Pulmonary Lining Fluid in Patients with COVID‐19: A Unique Perspective to Explore and Understand the Target Plasma Concentration of 292 ng/mL in Antiviral Activity
Source: Immun Inflamm Dis. 2024 Nov 15;12(11):e70075. doi: 10.1002/iid3.70075 (PMC11565441; doi:10.1002/iid3.70075)
Supplement: Supplementary file 2 — Supporting information. [file IID3-12-e70075-s002.docx]

**Table S1 Demographic and clinical characteristics of the patients**

| **Characteristic** | **Patient 1** | **Patient 2** | **Patient 3** | **Patient 4** | **Patient 5** | **Patient 6** | **Patient 7** |
| --- | --- | --- | --- | --- | --- | --- | --- |
| Age (years) | 99 | 73 | 91 | 77 | 80 | 92 | 71 |
| Sex | Male | Male | Male | Male | Male | Male | Male |
| Weight (kg) | 45 | 50 | 60 | 80 | 70 | 60 | 74 |
| Nirmatrelvir/  Ritonavir dose | Round 1:150mg/100mg  Round 2:300mg/100mg adjust to 150mg/100mg | 300mg/100mg adjust to  150mg/100mg | 300mg/100mg adjust to  150mg/100mg | 300mg/100mg | 300mg/100mg | 300mg/100mg adjust to  150mg/100mg | 300mg/100mg |
| Frequency | qd | qd | bid adjust to qd | bid | bid adjust to qd | bid | bid |
| Administration | Gastric tube injection | Gastric tube injection | Gastric tube injection | Gastric tube injection | Gastric tube injection | Oral administration | Gastric tube injection |
| Serum Creatinine^*^ (μmol/L) | 110 | 141 | 106 | 123 | 72 | 52 | 49 |
| eGFR^*^  (mL/min/1.73m^2^) | 56.9 | 45.4 | 60.3 | 52.6 | 96.8 | 137.0 | 154.2 |
| Combined use with CYP3A4 inhibitors | Omeprazole、Esomeprazole、Amiodarone | Amiodarone | Pantoprazole、Esomeprazole、Amiodarone、Voriconazole、Posaconazole | Esomeprazole、Fluconazole | Esomeprazole、pantoprazole、Voriconazole、Amiodarone | Esomeprazole | Fluconazole、Esomeprazole |
| Combined use with CYP3A4 inducers | Methylprednisolone | Methylprednisolone | Methylprednisolone | Methylprednisolone | Methylprednisolone | Methylprednisolone | NA |
| Combined use with CYP3A4 substrate | Lidocaine | Lidocaine、Midazolam | Baricitinib、Lidocaine、Midazolam | Lidocaine、Midazolam | Lidocaine、Midazolam | Lidocaine | Lidocaine |

Note: * Data on the first day of administration; eGFR,estimate glomerular filtration rate : Abbreviated MDRD study equation: *GFR* (mL/min per 1.73 m^2^ )=186×(*SCr*)^-1.154^ *(Age)*^-0.203^×(0.742 if female 1.210); NA:Not Applicable.
